# Warburg effect and translocation-induced genomic instability: two yeast models for cancer cells

**DOI:** 10.3389/fonc.2012.00212

**Published:** 2013-01-18

**Authors:** Valentina Tosato, Nana-Maria Grüning, Michael Breitenbach, Remigiusz Arnak, Markus Ralser, Carlo V. Bruschi

**Affiliations:** ^1^International Centre for Genetic Engineering and BiotechnologyTrieste, Italy; ^2^Cambridge System Biology Center, Department of Biochemistry, University of CambridgeCambridge, UK; ^3^Division of Genetics, Department of Cell Biology, University of SalzburgSalzburg, Austria

**Keywords:** aneuploidy, cancer, chromosome translocation, double-strand break, genome stability, pentose-phosphate pathway, Warburg effect, yeast model system

## Abstract

Yeast has been established as an efficient model system to study biological principles underpinning human health. In this review we focus on yeast models covering two aspects of cancer formation and progression (i) the activity of pyruvate kinase (PK), which recapitulates metabolic features of cancer cells, including the Warburg effect, and (ii) chromosome bridge-induced translocation (BIT) mimiking genome instability in cancer. *Saccharomyces cerevisiae* is an excellent model to study cancer cell metabolism, as exponentially growing yeast cells exhibit many metabolic similarities with rapidly proliferating cancer cells. The metabolic reconfiguration includes an increase in glucose uptake and fermentation, at the expense of respiration and oxidative phosphorylation (the Warburg effect), and involves a broad reconfiguration of nucleotide and amino acid metabolism. Both in yeast and humans, the regulation of this process seems to have a central player, PK, which is up-regulated in cancer, and to occur mostly on a post-transcriptional and post-translational basis. Furthermore, BIT allows to generate selectable translocation-derived recombinants (“translocants”), between any two desired chromosomal locations, in wild-type yeast strains transformed with a linear DNA cassette carrying a selectable marker flanked by two DNA sequences homologous to different chromosomes. Using the BIT system, targeted non-reciprocal translocations in mitosis are easily inducible. An extensive collection of different yeast translocants exhibiting genome instability and aberrant phenotypes similar to cancer cells has been produced and subjected to analysis. In this review, we hence provide an overview upon two yeast cancer models, and extrapolate general principles for mimicking human disease mechanisms in yeast.

## INTRODUCTION

Yeast (*Saccharomyces cerevisiae*) and human split around a billion years ago, therefore a plethora of cellular mechanisms have evolved in parallel. However, at the same time many fundamental processes remain strongly conserved, and thus yeast represents an efficient utility that can help to understand the molecular mechanisms underlying human disease physiology. Although many yeast models for studying cancer have been established (reviewed in Pereira et al., 2012), we here focus on two physiological processes that appear to be deeply similar between yeast and humans: (i) the reprograming of central metabolism during rapid cell growth [glycolysis, the pentose-phosphate pathway (PPP), amino acid metabolism, and respiration], bearing similarities to the Warburg effect in cancer cells (Bayley and Devilee, 2011; Cairns et al., 2011; Grüning and Ralser, 2011), and (ii) the bridge-induced translocation (BIT) system and its genetic and physiological consequences (Tosato et al., 2005, 2009; Nikitin et al., 2008; Rossi et al., 2010), which resemble and perhaps could simulate genomic instability of leukemia cells.

The majority of cancer research institutions around the world use yeast genetics as part of their research strategy and at least two of them are (or were) led by Nobel laureates who achieved their major honors for research accomplished in yeasts (Leland H. Hartwell and Paul Nurse, who together with Tim Hunt were awarded the 2001 Nobel prize in Physiology or Medicine for their work on the eukaryotic cell cycle). *S. cerevisiae* remains prominent in research of basics of eukaryotic molecular cell biology. Undisputedly, this yeast is a very advantageous system for this purpose: *S. cerevisiae* cells are rapidly growing and easy to handle, have a short cell cycle and use a large number (but not all) of the molecular genetic mechanisms known from multicellular organisms. Most importantly, yeast is the most highly developed system amenable to change its genome by genetic engineering, reintroducing precisely engineered genetic changes into the genome and to study the effects of those manipulations *in vivo*, in short, to do “reverse genetics.” This is in particular true for cell cycle regulation, mutagenesis, and DNA repair, and the suicide process of apoptosis, all of which have been found to be important for understanding the biology of cancer cells. To give an example and a quote, the motivation of Lee Hartwell to do cell cycle research in yeast was that he wanted to contribute to the understanding of cancer. He started his Nobel lecture with these words: "My research career has been motivated by a desire to understand cancer. Each time I have identified an intriguing aspect of the cancer problem, I found that it could be approached more effectively in the simple eukaryotic cell, *S. cerevisiae*, than in the human cell” (Hartwell, 2004). The key point is to mimic, if possible, the pathological changes observed in cancer cells in yeast cells and then to manipulate these model phenocopies in order to try to reduce the effects of those changes. Examples would be the loss of cell cycle checkpoints in cancer cells and the loss of pathways to enter the apoptotic program.

### COMPARATIVE GENOMICS

The prediction that nearly half (∼3,000) of all yeast genes would have structural or functional homologs in the human genome, prompted many comparative genetic studies between the yeast and mammalian cell systems. Indeed, several yeast orthologs exist of human genes considered tumor suppressors important for tumor initiation and/or progression. For other genes, like the human p53 tumor suppressor and cell cycle checkpoint gene, even if there is no direct yeast ortholog itself, an analogous signal transduction pathway in which it participates does exist. The human protein can be expressed in yeast, where the mutations occurring in this gene and their phenotypic consequences can be studied much easier. In this way, the mutational spectrum of p53 was determined and found to be identical to the one found in human cancers (Brachmann et al., 1996; Inga et al., 1998; Schlichtholz et al., 2004). Since the genetic system of yeast allows for the selection of specific types of mutants, for instance dominant negative mutations, the spectrum of dominant negative mutations of p53 obtained in yeast was then found identical to the mutational spectrum in cancers (Brachmann et al., 1996).

New anti-cancer drugs that ideally should interfere with the special pathological processes of cancer cells without harming normal cells are being tested in yeast cells mimicking “cancer-like” genetic (mutants defective in checkpoints) or environmental (cells under severe oxidative stress) conditions. The efficacy of these drugs often depends on a “synthetic lethal” effect. For instance, as a driving mutation inactivates a certain repair pathway, the drug inhibits the only other remaining parallel repair pathway in the cancer cell (Bjornsti, 2002; Hartwell, 2004). As outlined by Hartwell, these strategies have already proved very valuable for broadening the understanding of cancer development and treatments given to patients. As example, our understanding of the molecular mechanism of action of cancer drugs which inhibit topoisomerase II or the proofreading activity of DNA polymerases has improved greatly using a panel of 70 yeast strains that are defective in exactly those highly conserved cell cycle, checkpoint, or DNA repair functions which are also found in clinical cancer specimens (Hartwell, 2004). Therefore, therapies can be developed and improved, if the relevant biochemical defect of the cancer in question is determined. A more exotic approach is to use yeast cells or substances derived from yeast cells as a cancer cure (Ghoneum and Gollapudi, 2004; Liu et al., 2009) that is, however, not the topic of this review.

### GENOME STABILITY AND MAINTENANCE

An important contribution of yeast research to our understanding of cancer arose from genome-wide screenings for mutations that decrease genomic stability (Yuen et al., 2007; Stirling et al., 2011). This concerns missegregation of chromosomes resulting in aneuploidy, chromosome mutations like translocations, inversions and deletions, and also point mutations. Both the mitochondrial and the nuclear genomes were considered for these lines of research. Also included in these attempts are investigations of aging cells that were found to show an increased level of genetic instability. In the last few years, these attempts were supported by an increasing number of whole genome sequences of human tumor cells. These sequences identified hundreds of mutations in those tumors and have resulted in the identification of new cancer-relevant genes, including CIN (chromosomal instability) alleles (Bignell et al., 2010). We believe that the genetic changes that give rise to the genetic instability of tumor cells may provide the key to tumor cell sensitivity (Hartwell et al., 1997).

The occurrence of genomic instability is an almost universal marker of cancer cells, but it is less clear if mutations leading to genomic instability are the most important early “driver” mutations which clonally initiate cancers (Michor et al., 2005). This view is now enlarged by the findings of Marie Hardwick in yeast (personal communication) showing that in a majority of all the deletion mutants which she looked at in their yeast collection, secondary mutations were quite unexpectedly (but rapidly) selected and occurred in the strains as they were distributed out. These secondary mutations were in multiple genes and also represented multiple alleles within the same gene (example: *WHI2*). This genomic instability could be due to the selective forces that apparently work in a genome where a functional gene has been lost.

It is also not clear if a single chromosomal translocation event can be sufficient to trigger the complex chain of events that we call tumor progression. This is the case in certain leukemias, the most well known of which are chronic myelogenic leukemia (CML) and acute myeloid leukemia caused by the translocation event of the Philadelphia chromosome (Sherbenou and Druker, 2007). At the present, even if the earliest direct demonstrations of the role of chromosomal translocations as causative agents of tumors were found in liquid tumors, there are nevertheless numerous examples, even in epithelial tumors, of the pathogenic effect of these translocations in solid tumors as well.

We would like to ask the question as to “what would conceivably be the phenotype of yeast cells that can serve as a model for human cancer cells?” To many people’s opinion, unrestricted growth is the most problematic phenotype of cancer cells. Normal wild-type yeast cells recapitulate this phenotype: on rich media, cells multiply and the biomass grows until nutrients, or one essential nutrient, are used up. However, wild-type cells are able to respond to the level of all essential nutrients, and to many other conditions (for instance, the presence of an alpha mating partner in an a-cell) in an ordered and life-promoting fashion. These responses work via signal transduction pathways or rather a network of such signal transduction pathways that regulates the cell cycle. These mechanisms can stop the cell cycle at junctions that were termed “checkpoints” by Hartwell and his coworkers (Hartwell and Weinert, 1989; Weinert, 1997). The final phenotypic outcome of these wild-type checkpoint mechanisms is cell cycle arrest (for instance in response to DNA damage), repair, and if repair does not take place, either apoptotic cell death or trans lesion DNA synthesis resulting in a permanent damage to the genome, and hence genomic instability. These pathological forms of growth and cell division were not observed frequently in yeast mutants, but they are hallmarks of human cancer cells.

#### Early yeast models for cancer signaling, the RAS gene

In one of the first valid examples of “cancer phenotypes” in yeast, the oncogenic point mutation Ha-ras-val12 was compared with a homologous mutation in a closely related yeast gene, *RAS2*-val19. It is well known that this single point mutation can transform mouse 3T3 cells from an immortalized but harmless cell line into a highly cancerogenic line leading to numerous cancerogenic *foci* in cell culture and to cancers if transplanted into immune deficient mice (Weinberg, 1983). The same mutation in yeast (*RAS2*-val19) renders yeast cells into being insensitive to the starvation signal(s) and prevents the synthesis of reserve carbohydrates. Eventually, this causes cell death during starvation (in particular nitrogen starvation), a very short mother cell-specific life span even in the presence of nutrients, creates oxidative stress and increases apoptotic death (Tatchell et al., 1985; Toda et al., 1985). Biochemically, the two homologous mutant genes in yeast and human cells render the small G protein RAS insensitive to regulatory proteins (GAPs and GEFs), keep it in a permanently activated state and therefore permanently activate the ensuing signal transduction cascade. Interestingly, although the downstream kinases are different in yeast and human cells, the observed changes resulting from the activating ras mutation are similar in both systems. If the human Ha-ras-val12 mutation is tested in an otherwise wild-type cell, it leads to hypermitogenic arrest and apoptosis, just as in the yeast cell (Serrano et al., 1997). Only if other typical mutations of cancer cells are present, the murine cells are transformed to acquire cancerous growth. This example shows what can be expected from yeast cells that model cancer cells and which cancer phenotypes can be studied in yeast. In our view, two of the most telling and pathogenetically relevant phenotypes of cancer cells are their genomic instability and the remodeling of metabolism to a hypoxic-like state although oxygen is present in those cells at levels comparable to wild-type non-cancerous cells. Little or nothing is presently known about the interrelationship of these two phenotypes. The latter one is also known as the Warburg effect and has recently experienced a renaissance of intensive investigations among cancer researchers. Genomic instability occurs in the majority if not in all cancer cells, but the role and history of subsequent steps of genomic instability during tumor progression is hard to study because early stages of cancers are usually not available in clinical samples. It is unclear how and why a final step in tumor progression results in a highly aneuploid but finally stable endpoint and what the biochemical commonalities are between those vastly different cancer cells, which are all highly aneuploid. For those reasons, a number of groups have tried to define mutations in genes which are normally responsible for genomic stability and lead to strong mutator phenotypes resulting in chromosome loss or gross chromosomal rearrangements (GCR) in certain mutants. It is now possible but very difficult to perform such a study in human cells (Paulsen et al., 2009). However, the yeast genetic system has been exploited for this purpose and has led to a comprehensive set of both non-essential (Yuen et al., 2007) and essential genes (Stirling et al., 2011) which, when mutated, contribute to genomic instability. The yeast system has the additional advantage to define whole sets of genes and physiological pathways through interaction networks, which also contributes to genome maintenance. The endpoints used for the genome-wide screening of both the yeast deletion collection and several collections of conditional mutations in essential genes were (i) loss of a centromeric chromosome fragment and screening for ade2-mutant colony color; (ii) screening for bi-maters; (iii) screening for a-like fakers; and (iv) screening for GCRs by simultaneously scoring forward mutation to canR and loss of *URA3* on 5-fluoroorotic acid (5-FOA) (Stirling et al., 2011). A total of 692 genes was identified in functional classes (GO terms) that are highly plausible based on prior knowledge of genome maintenance (mitosis, replication, repair, DNA modification, telomere maintenance, transcription, RNA processing, nuclear transport, and proteasome) or define peripheral functions, like iron-sulfur cluster biosynthesis (Veatch et al., 2009). Most importantly, some of the most central and most highly conserved of those genes have human orthologs, mutant alleles of which were found in tumor specimens – among them, *SGS1* (human genes *BLM* and *WRN*, repair helicase), *MRE11* (*MRE11A*, the gene of a syndrome related to ataxia telangiectasia),* DUN1 *(*CHK2*, one of the genes of familial Li–Fraumeni syndrome, coding for a cell cycle checkpoint protein), and *BUB1 *(*BUB1*, frequently mutated in colorectal cancer). These findings are proof of principle for the usefulness of this approach to study genome instability in yeast as an avenue to understand genome instability in cancers. The consensus among the authors in this field is that genome instability can probably be a primary cause creating the other mutations found in cancers and constituting the tumor progression sequence. A similar conclusion was reached by analyzing clinical data with a mathematical model in light of the “two hit” hypothesis (Michor et al., 2005). The activation of error prone DNA synthesis, which then indirectly leads to more mutations including point mutations, is another important consequence of genome instability (Daee et al., 2010). This can happen both by activating error prone DNA polymerases that exist in all cells for different purposes, and also by producing mutant forms of the replicative DNA polymerases, which for instance have lost their proofreading exonuclease functions (Daee et al., 2010).

We believe that BIT in diploid yeast cells (Tosato et al., 2005), which leads to unstable genomes, can be a valid model system to study the role and the physiological consequences of genomic instability. This system consists of the artificial induction of chromosome translocation based upon the DNA transformation of wild-type yeast cells with a linear, double-stranded DNA molecule (cassette) obtained by PCR. The DNA cassette has the two ends with a sequence homologous to two different chromosomal sites of the genome and flank a positively selectable marker such as *KAN*^*R*^ or *HYG*^*R*^. The integration of the two free DNA cassette ends at their homologous site by homologous recombination, forms a DNA bridge between two different chromosomes, that is, a chromosome translocation between them. This event can be selected screening the cells for the stable appearance of the phenotype conferred by the selectable marker carried by the cassette.

The connection between unstable genomes and metabolic remodeling can be studied in those cells that have undergone BIT translocation. Indeed, in these cells, a complex genomic rearrangement is triggered after the primary BIT event, leading to a general status of gene de-regulation that slowly settles down, selectively remodeling the metabolism according to the environmental conditions of growth, in what is called the adaptation phase.

## STUDYING YEAST PHYSIOLOGY TO EXPLAIN THE WARBURG EFFECT OF CANCER CELLS

The leading biochemist Otto Warburg described as early as in the 1920s that tumor tissue ferments at the expense of respiratory activity (reviewed in Warburg, 1956). He speculated that defects in mitochondria are thus a cause of cancer. From today’s perspective, it is known that most cancer cells, with the important exception of oncocytoma (Mayr et al., 2008), possess a functional respiratory chain. However, most of them show increased uptake of glucose (this property is explored in imaging to stain cancer tissue using the glucose analogous probe 2 fluoro-deoxy-glucose (2FDG; Kurtoglu et al., 2007)), although many cancers have reduced activity of oxidative phosphorylation (Ferreira, 2010; Cairns et al., 2011). From the historical view of metabolism as a producer of energy and intermediates, this behavior is counterintuitive as mitochondrial respiration is more efficient in energy production compared to anaerobic glycolysis, and the rapid growth of tumors has a high demand for energy (Cairns et al., 2011; Gruning et al., 2011). Research of the recent years, involving yeast as central model now indicates, that metabolic integrity and homeostasis of the system could explain the necessity of wasting energy, and reconfiguring metabolism when growing rapidly.

Yeast recapitulates features of the Warburg effect. At maximum growth speed, *S. cerevisiae *strongly prefers fermentation to respiration. Hence under conditions where sufficient nutrients are available, energy (in form of ATP) appears not to be a limiting factor for rapid proliferation. After entering the stationary phase, yeast growth is slowed and the demand for energy declines. Interestingly, it is exactly this point where respiration becomes an important source of energy (van Dijken et al., 1993). This important physiological parallelism between most cancer cells and yeast has prompted a vigorous research in this area.

The yeast *S. cerevisiae* is a very useful tool in studying the Warburg effect, as respiratory metabolism can be induced or repressed easily via switching the carbon source (De Deken, 1966; Crabtree effect, described by H. G. Crabtree in the 1920s; Crabtree, 1928). Although they have roughly the same energy content and are both fermented, glucose represses respiration, but galactose does not. Ruckenstuhl et al. (2009) used this yeast property to investigate the effects of respiratory bursts on apoptosis, and the impact of free radicals on this process. Inhibition of respiration, or free radical scavenging conferred a survival advantage during seeding and early development of yeast colonies (Ruckenstuhl et al., 2009). Similarly, cancer cells are reactive oxygen species (ROS) sensitive, and might profit from anti-oxidant therapies (Perera and Bardeesy, 2011).

Recent advances in understanding the Warburg effect in cancer and yeast came from investigation of the enzyme pyruvate kinase (PK), which was recognized as a cellular coordinator of respiration and of the anti-oxidant system. It has been reported that exchanging the human PK isoform pyruvate kinase muscle isozyme 2 (PKM2) with its constitutive isozyme PKM1 dramatically slows growth of xenograft tumors, and reactivates respiratory metabolism (Christofk et al., 2008). In this context, it was assumed that PKM2 is specific to proliferating tissue, found only in the embryo or in cancer cells. The lack of other cancer-specific metabolic enzymes thus placed PKM2 center stage for research on cancer cell metabolism (Bayley and Devilee, 2011; Cairns et al., 2011; Hamanaka and Chandel, 2011). However, a PKM2 cancer specificity could not be confirmed in follow up studies, indeed most adult tissues express PKM2 as their predominant PKM form independent of whether they are cancerous or healthy (an exception is, however muscle, which expresses PKM1; Bluemlein et al., 2011). The interpretation of PKM2 being cancer-specific thus potentially resulted from using mouse muscle tissue as non-cancer control in Western blot experiments (Christofk et al., 2008). Despite this setback, however, PK emerged as a central regulator of glycolysis, with immense importance for cancer (Bayley and Devilee, 2011; Chaneton and Gottlieb, 2012). Yeast models helped in understanding the global function of this enzyme.

**FIGURE 1:**
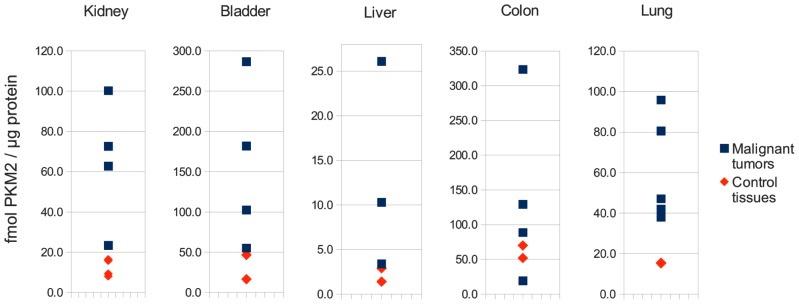
**Pyruvate kinase (PK) activity as regulator of anti-oxidant metabolism.** Low activity of PK, found in cancer and in respiring yeast, leads to increased flux of the pentose phosphate pathway. Increased PPP activity is required for maintaining the redox balance under conditions with high ROS load: reduced NADPH is required as redox-power for anti-oxidant enzymes, and PPP activity stimulates the anti-oxidative gene expression program.

These results might further help to solve a seemingly paradox about PK activity in cancer cells: as discussed by Diaz-Ruiz et al. (2011), the inhibition of PKM2 seems contradictory in respect to the high glycolytic flux and increased lactate excretion measured in cancer cells, since an inactive PK would severely impair cell energy production in the cells that depends mainly on glycolysis for ATP synthesis. Indeed however, studying PKM2 expression using absolute quantitative mass spectrometry reveals that PKM2 levels are thoroughly higher in cancer cells than they are in matched control tissues (as determined in Bluemlein et al., 2011; please see **Figure 1** for an illustration). Thus, although *PKM2* is not to 100% active in cancer, its overall activity might still be higher as it is in the corresponding control tissue. Indeed, recent investigations show that *PKM2 *knock-down increases flux of the TCA cycle and amino acid metabolism also in cancer cells, indicating that PKM2 is of considerable residual flux in tumors (Chaneton et al., 2012).

**FIGURE 2:**
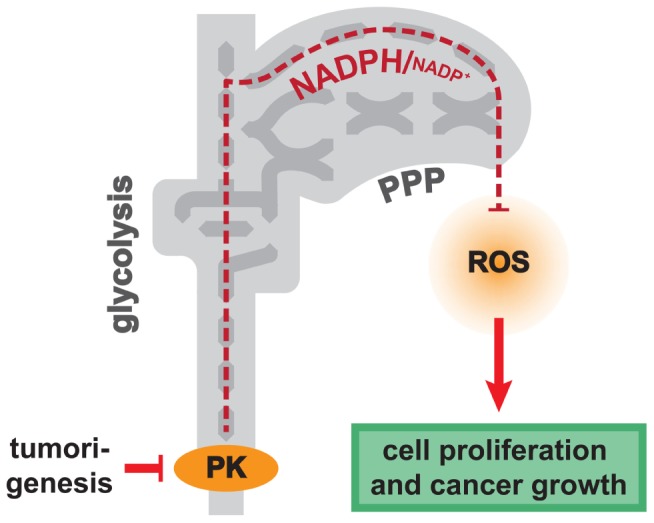
***PKM2 *is up-regulated in human cancer.** Illustration of the absolute concentrations of PKM2 in several cancer tissues and matched controls, as determined by liquid chromatography-multiple reaction monitoring (LC-MRM) as described in (Bluemlein et al., 2011). PKM2 levels are clearly increased in cancer biopsies compared to the controls.

Similar to PKM proteins in mammalian cells, yeast possesses two paralogous PK genes, *PYK1* and *PYK2* that encode for enzymes with related properties. *PYK1* encodes for the predominant PK isoform when cells grow on glucose media, whereas Pyk2p has lower specific activity and is induced under respiring conditions (Boles et al., 1997). Creating yeast strains with different PK activities (by ectopic expression of either Pyk1p or Pyk2p at both high and low level in a Δ*pyk1*Δ*pyk2* strain) we found that a reduced activity of this enzyme is sufficient to increase oxygen uptake and respiratory activity. Unexpectedly however, strains with lower PK activity exhibited an increased resistance to several oxidants. Moreover, although respiring at higher rates, these cells did not show an increase in the concentration of superoxide and hydrogen peroxide, nor did they display features of oxidative stress (Gruning et al., 2011). This indicated that low PK activity does not only lead to increased respiration, it also causes an increase in the anti-oxidant capacity. This physiological reconfiguration eventually compensated for the increased ROS generation during oxidative metabolism (**Figure 2**).

Pyruvate kinase converts phosphoenolpyruvate (PEP) to pyruvate, a reaction which yields one molecule of ATP (Fraenkel, 1986). The substrate PEP is a highly polar sugar phosphate, and accumulates in yeast and *E. coli *when PK activity is low (Emmerling et al., 2002; Gruning et al., 2011). It has been reported that PEP can interfere with more than one reaction of glycolysis, including phosphoglycerate mutase, glucokinase, phosphoglucoisomerase, phosphofructokinase, aldolase, and triosephosphate isomerase (TPI; Ogawa et al., 2007; Fenton and Reinhart, 2009; Vander Heiden et al., 2010). Interestingly, inhibition of the latter was sufficient to increase resistance to oxidants in yeast and *C. elegans*. The increase in stress resistance can be attributed to increased metabolite levels in a metabolic pathway parallel to glycolysis, the PPP (Ralser et al., 2006, 2007). The PPP shares several metabolites with glycolysis, and plays a pivotal role in the oxidative stress response. First, this pathway can quickly and dynamically increase in activity to suffice the increased need for the redox co-factor NADPH upon an oxidative burst (Ralser et al., 2009). Second, it is involved in the induction of the anti-oxidant gene expression program (Kruger et al., 2011). It appears that TPI feedback inactivation by PEP is required for the increase in stress resistance of the PK mutants, as cells expressing a mutant human TPI allele (TPI^Ile170Val^) that is largely robust to PEP inhibition do not show the PK dependent increased resistance to oxidants. *Vice versa*, in cells with low PK activity, deletion of the first enzyme of the PPP, glucose-6 phosphate dehydrogenase (*ZWF1*) causes increased ROS levels, protein oxidation, and mitochondrial damage (Gruning et al., 2011). In sum, a reduction of PK activity increases the flux of the PPP protecting against oxidants, and the feedback inhibition of TPI by the PK substrate PEP is crucial for this adaptation.

Although respiring at moderate rates, also cancer cells suffer from high ROS load (Pelicano et al., 2004; Chandra and Singh, 2011; Israel and Schwartz, 2011; Perera and Bardeesy, 2011). It is assumed that the majority of these ROS are side products of the high metabolic activity of cancer cells, especially beta-oxidation of fatty acids, and the activity of NADPH oxidases (Pelicano et al., 2004; Cairns et al., 2011). However, this information has a high degree of uncertainty, as a reliable genome-wide quantification of ROS contributions in cancer is lacking till the present day. Nonetheless, maintaining the redox balance appears to be a major issue for mammalian tissue, indicated by the high concentration of the anti-oxidant peptide glutathione, which exceeds the cellular ATP level by an order of magnitude (Meister and Anderson, 1983). In tumors, PKM2 seems to fulfill a similar role in anti-oxidant defense as discovered for yeast *PYK* genes. The low activity of PKM2 in lung cancer cells leads to a higher activity of the NADP reduction in the PPP, and to increased anti-oxidant defense. Expressing of a oxidation-resistant PKM2 mutant in xenograft tumors reduced the activity of the PPP, and markedly slowed tumor growth (Anastasiou et al., 2011).

Overall, these observations indicate that the balancing of the metabolic network (and so maintaining the redox state and metabolite homeostasis) might be more difficult to achieve for rapidly proliferating cells than to guarantee a sufficient supply with ATP. Understanding this principle can be very valuable for developing anti-cancer therapies. For instance, one could imagine inducing a ROS boost into cancer cells for making them vulnerable to chemotherapeutics (Perera and Bardeesy, 2011). The broad experimental possibilities offered by yeast are invaluable help in deciphering these complex questions.

Interestingly, recent results from several laboratories reveal that a redirection of central carbon metabolism by PK does not only change redox metabolism, but is also important for amino acid metabolism (Bluemlein et al., 2012; Chaneton et al., 2012; Kung et al., 2012; Ye et al., 2012). In yeast, a change in the activity or expression level of PK causes a strong reconfiguration of the entire amino acid profile, with seven amino acids (arginine, aspartic acid, histidine, lysine, threonine, valine, and serine) being present a lower concentration, and two amino acids (glutamine and glutamate) being increased when PK activity is low (Bluemlein et al., 2012). Hence, PK seems to link the generation of energy within central metabolism, and the metabolism around energy consumption at the level protein biosynthesis.

In mammalian cells, the function of PKM2 in regulating serine biosynthesis has been studied in detail, and reveals a feedback control system which controls the levels of free amino acids. It has been found that serine is an allosteric activator of human PKM2, and that overall PK activity is reduced when cancer cells are deprived of this amino acid (Chaneton et al., 2012; Ye et al., 2012). At the same time, the glycolytic block caused by reduced PKM2 activity feeds back into serine biosynthesis, preventing serine deprivation during cancer formation (Chaneton et al., 2012). Consistently, in human thyroid follicular adenoma, the expression of the serine-biosynthetic enzyme serine hydroxymethyltransferase (SHMT1) is increased compared to healthy control tissue, and correlates with the absolute PKM2 expression level (Bluemlein et al., 2012). In addition, small molecule activation of PKM2 induces serine auxotrophy in cancer cells, indicating that this control mechanism could be exploited for therapeutic purposes (Kung et al., 2012).

In sum, studies in yeast led to the discovery of redox state control by the enzyme PK (Gruning et al., 2011). This mechanism appears to be of importance for the progression of lung cancer cells, but potentially other cancer types as well (Anastasiou et al., 2011). PK further moonlights to the regulation of protein biosynthesis, and amino acid metabolism in yeast and human cells (Bluemlein et al., 2012; Chaneton et al., 2012). Serine appears to be central for this regulatory mechanism, as it can act as allosteric activator of PKM2 and hence report the concentration of free amino acids to central carbon metabolism (Chaneton et al., 2012; Ye et al., 2012). The PK enzyme is thus a central player in coordinating cellular metabolism. Yeast turned out to be a very effective model in studying the interplay of the involved metabolic pathways.

## SPONTANEOUS AND INDUCED CHROMOSOMAL TRANSLOCATIONS

One of the best possibilities offered by the yeast system to model gross genetic alterations known to induce well-characterized cancer forms in humans, is the BIT, bridge-induced chromosomal translocation. Indeed, this technology induces the formation of a translocated chromosome exploiting the yeast natural homologous recombination system (HRS) between the two ends of a DNA bridge molecule harboring a positively selectable marker (i.e., *KAN*^*R*^). This type of chromosomal aberration has been since long time connected to the insurgence of forms of tumors, like the renowned Philadelphia chromosome, resulting from a translocation between chromosome 9 and 22 in humans, leading to CML. Several other chromosomal translocations are known to promote cancer and their molecular mechanisms of occurrence can be studied efficiently in yeast, using the inducible BIT system. In the following part, chromosomal translocations and BIT will be deeply analyzed with respect to their cellular consequences leading ultimately to cancer.

Chromosomal translocations are rare cellular phenomena in which two chromosomes are interacting with each other either by physical fusions or by copying one chromosome’s fragment on another. Depending on the nature of these interactions, chromosomal translocations can have reciprocal or non-reciprocal configuration. Translocations might pass unnoticed by the cell, bringing no consequences; however, in the majority of cases, a translocation’s onset has a tremendous impact, limited not only to the single cell, but also to the organism as a whole. Chromosomal translocations yield a variety of effects ranging from distorted transcription patterns to cell death due to increased apoptosis. In multicellular organisms, chromosomal translocations can be related to a systemic death observed in human malignancies, in particular hematological or mesenchymal cancers. As a result, translocations can also be useful markers in the diagnosis of liquid and solid tumors (Herve et al., 2011; Klemke et al., 2011). This fact greatly increases interest in the investigation of all chromosomal translocations aspects: origins, causes, outcomes, and clearly – their association with genetic diseases. Despite the growing number of laboratories working on these subjects and the great efforts made by investigators all over the world, the topic of chromosomal translocation is still not fully covered and the mechanistic molecular factors that elicit these GCRs are still object of investigation. Progresses are greatly impaired by the rare occurrence of spontaneous translocations, in particular in mammalian cells, by the broad panorama of secondary rearrangements and by a lack of effective detection techniques.

Spontaneous chromosomal translocation can arise either from spontaneous recombination between repeated elements dispersed through a genome or from the free DNA ends originated from double-strand breaks (DSBs), stalled replication forks or dysfunctional telomeres (Jinks-Robertson and Petes, 1986; Loidl and Nairz, 1997; Richardson et al., 1998). Although DSBs are an extreme threat to the cell, they are crucial for its existence and happen very frequently in a programmed manner as a part of specific life cycle processes such as meiosis, mating type switching in fungi or V (D) J recombination during immunoglobulin and T-cell receptors maturation (Bassing et al., 2002; Zhang et al., 2011). DSBs can appear as a result of cellular processes like DNA replication, through single-strand nicks, or elevated levels of ROS, but they can be also induced by exogenous factors. Cell exposition to DSBs-inducing agents (e.g., ionizing radiation, ROS, viruses, some chemotherapeutic drugs, and more) greatly enhance the probability of spontaneous translocations and teratogenicity, as even a single DNA break in the cell can result in GCRs (Kolodner et al., 2002). There exist two major, different DSB repair pathways: homologous recombination (HR) and non-homologous end joining (NHEJ), and both of them can give rise to translocations. HR can occur by means of three sub-pathways: gene conversion (GC), break induced replication (BIR), and single-strand annealing (SSA). For a detailed review of these repair pathways in mammalian and yeast cells the reader is referred to (Aylon and Kupiec, 2004). Genome rearrangements, by definition, are not beneficial for the cell and will be actively prevented by various mechanisms. For example, the choice of the correct repair pathway (HR or NHEJ) at the right moment of the cell cycle is fundamental for the suppression of translocation (Branzei and Foiani, 2008). In budding and fission yeast, HR is the dominating pathway for DSB repair, and NHEJ seems to be restricted only to the G1 phase. In fact, experiments indicate that NHEJ mutants of *S. cerevisiae* are resistant to ionizing radiation, whereas HR mutations severely compromise survival (Siede et al., 1996; Manolis et al., 2001). In mammalian cells the situation is opposite. NHEJ is the dominating repair pathway employed during the entire cell cycle, whereas HR is restricted to the S phase (van Gent and van der Burg, 2007; Shrivastav et al., 2008). In effect, NHEJ pathway was originally identified in mammals, and later its elements were discovered in bacteria and yeasts (Moore and Haber, 1996; Doherty et al., 2001). The prevalence of the HRS in budding yeast suggested its exploitation in the production of “*ad hoc*” translocations in *S. cerevisiae*, as described in the following paragraphs.

The broad spectrum of the effects of translocations and their complex involvement into cancerogenic processes are the reason why chromosomal translocations are so intensively studied. Neoplastic transformation is associated to reciprocal or non-reciprocal translocations that can lead to altered expression of proto-oncogenes and loss of heterozygosity (LOH) of tumor suppressor genes. Proto-oncogenes have their own homolog in budding yeast (the most popular is *SAS3*, ortholog of *MOZ*) as also do tumor suppressors (the yeast genes *TEP1*, *FSH1*, *HNT2*), with the renowned exception of *TP53.*

The genetic mechanism through which chromosome translocations elicit the onset of certain tumors is the fusion of the coding sequence of two non-contiguous genes located at the translocation site, on different chromosomes, with the consequent expression of a novel hybrid protein able to disrupt the correct control of cell proliferation. Usually, two major groups of genes are involved into neoplastic transformation as an indirect result of chromosome translocation: tyrosine kinases and transcription factors. Oncogenic mechanisms of chimeric proteins results from the cancer-promoting nature of such proteins or by disruption of another gene regulation system. Novel gene fusions caused by DSB repair are responsible for around 20% of human cancer morbidity. Until now, 337 genes were identified in 358 gene fusions (Mitelman et al., 2007). These numbers are rapidly increasing due to development of rapid sequencing methods and constantly growing microarray databases. Identification of gene fusions gains remarkable importance as a diagnostic and prognostic marker (Prensner and Chinnaiyan, 2009). The role of chromosomal translocations in neoplasia is so significant, that a specialized database of chromosomal aberrations and gene fusions in cancer has been created. This database can be accessed at: http://cgap.nci.nih.gov/Chromosomes/Mitelman.

However, as mentioned before, one of the main problems that researchers have to overcome is the extremely low frequency of spontaneous translocations arising either in mammals or in model systems. This negative aspect of the parallel between human cancer cells and yeast could be overcome by the induction of chromosome translocation events in both cellular systems with mutagenic agents. Unfortunately, any mutagenic process utilized in mammalian cells would require the appearance of a strong, detectable mutant phenotype to allow the selection of those cells that have undergone a translocation event, and this would occur very slowly and would be difficult to select for. On the contrary, with the model yeast cells, given the possibility to manipulate an almost endless number of them and to intervene more directly on their genome, this can be achieved rather easily. In order to increase the events to reach statistically significant numbers, various methods were developed for induction of chromosomal translocations. Generally, these methods are based on two major principles: artificial induction of DSBs within the desired regions sharing strong homology, or recombination between special elements catalyzed by site-specific recombinases. In both cases, a time-consuming molecular engineering of the sites selected for the translocation, is necessary prior the induction of the translocation event. Recently, a third methodology of induction of chromosomal translocation has been developed. This method, BIT, allows the generation of non-reciprocal translocations in mitosis without pre-modifications of the genome, exploiting the natural HRS of *S. cerevisiae*. The phenotypic changes of yeast cells after a BIT translocation event seem to mimic closely the oncogenic transformation of mammalian cells (Tosato et al., 2005). The most common methodologies to induce GCRs and their implications are discussed extensively in the next sections.

## ADVANTAGES AND DRAWBACKS OF THE MAIN MOLECULAR SYSTEMS TRIGGERING TRANSLOCATIONS

Several strategies to introduce DSBs by artificial means, ensuing in chromosome translocations were developed in the last 15 years. Transformation of yeast cells with a chromosomal fragmentation vector (CFV) resulted in the gain of a chromosomal fragment (CF) with or without the loss of the targeted chromosome, following DSB processing by break-copy duplication (Morrow et al., 1997). The discovery of fragmental duplication in yeast led to demonstrate, 1 year later, that a chromosomal DSB produced by the HO endonuclease could be repaired by BIR, producing NHEJ-mediated reciprocal translocation (Bosco and Haber, 1998). Later on the HO system has been utilized for the production of a DSB on two chromosomes, ensuing in reciprocal translocations by NHEJ (Yu and Gabriel, 2004) after the repair of the broken chromosomes by SSA (Liddell et al., 2011). Other meganucleases, such as I-SCEI, can be used to generate DSBs in higher organisms promoting translocations with a frequency of 1–4% (Egli et al., 2004). More recently, the cre site-specific recombination-based system producing reciprocal translocations at pre-engineered loxP sites has been developed to study speciation in yeasts (Delneri et al., 2003) and successively improved to minimize the occurrence of unwilled, secondary rearrangements (Carter and Delneri, 2010). Finally, transposable elements are regularly utilized to produce chromosomal manipulations with variable efficiencies. Among all these systems, transposons-related methodology are mostly exploited for the development of new variety of plants (Yu et al., 2012), the cre-lox is used to generate animal models of human cancers (Buchholz et al., 2000; Rabbitts et al., 2001; Forster et al., 2005; Yu et al., 2010) although meganuclease-related methods are also utilized to produce DSBs in malignant cell lines (Cheng et al., 2010; Kitao et al., 2011). However, all these experimental systems need preceding modifications of the genome in order to produce translocations. Moreover, almost always they result in reciprocal translocations. It was therefore necessary to develop a simple system that triggers translocations without any prerequisite for strain engineering, without the assistance of any cloned exogenous/endogenous enzyme and that allows also the simultaneous recovery of events such as non-reciprocal translocations, telomeric fusions and deletions usually occurring during neoplastic transformations. For these reasons the BIT methodology was developed.

## BIT: BRIDGE-INDUCED TRANSLOCATION

### THE BIT SYSTEM

**FIGURE 3:**
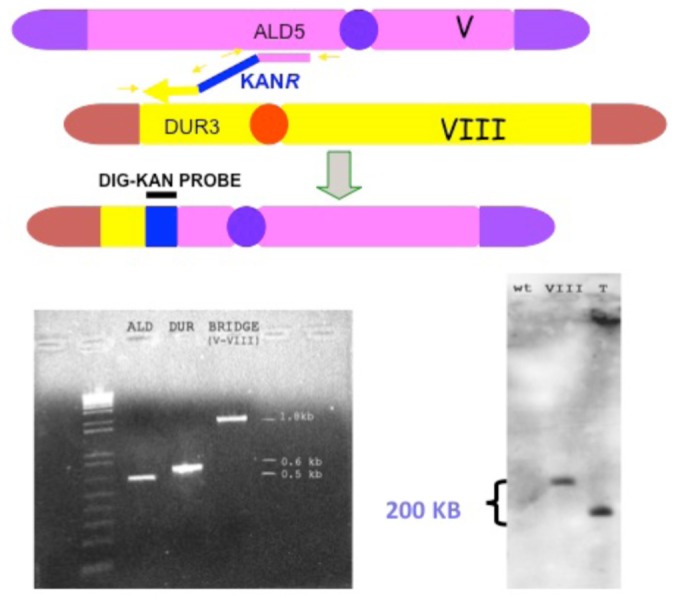
**Schematic representation of a BIT chromosome translocation event induced in the yeast *S. cerevisiae*, and its molecular verification by PCR and Southern blot analysis.** BIT translocation designed between the *ALD5 *locus on chromosome V and *DUR3 *on chromosome VIII and obtained by transformation with a linear double-stranded DNA cassette having the two extremities homologous to the two *loci*, flanking the positively selectable marker *KAN*^*R*^. The translocation between the two top chromosomes, catalyzed by the DNA cassette functioning as a bridge, produces the translocated chromosome below the big gray arrow. Verification of the correct chromosome translocation by gel electrophoresis analysis of PCR amplification (bottom, left) of the two DNA junctions at the *ALD5 *and* DUR3 loci *(between primers indicated by the two small yellow arrows on the right and the left, respectively), lanes ALD and DUR of the gel. Verification of the formation of the DNA bridge between the two chromosomes by PCR amplification of the region between the two external primers indicated by the two small external yellow arrows, lane BRIDGE of the gel. Bottom, right: Southern hybridization with a DNA probe corresponding to the *KAN*^*R*^ gene, of a contour-clamped homogeneous electric field (CHEF) electrophoresis spread of chromosomes from a wild-type strain (lane wt), a strain with chromosome VIII previously marked with *KAN*^*R*^ (lane VIII) and a strain subjected to BIT translocation at the same *loci* (lane T).

The system consists in the production of selectable translocation-derived recombinants (“translocants”) generated at desired chromosomal locations in wild-type yeast strains transformed with a linear DNA cassette carrying a selectable marker (i.e., KanR) flanked by two DNA sequences homologous to two different chromosomes (Tosato et al., 2005; **Figure 3**). The bridge, which is obtained exploiting the endogenous HR machinery of the yeast cell, is obtained with a variable efficiency (typically from 2 to 15%) depending on the length of the homologies, the secondary structures, and the base composition of the target regions, and the strain’s genetic background. This last variability is probably due to a different extension of the rDNA region of chromosome XII that may act as recombination hotspot. It was demonstrated that the resulting translocation is non-reciprocal, that it occurs with similar efficiency between heterologous and homologous chromosomes (Tosato et al., 2009) and that it is usually associated with aneuploidy (Rossi et al., 2010). Effectively, we verified that several other GCRs leading to LOH, such as intrachromosomal deletions, DNA duplications, unspecific translocations due to micro-homology, arose after transformation with the linear cassette.

### GENERAL CELLULAR EFFECTS OF BIT TRANSLOCATION

As a consequence of a single translocation event produced via BIT, the yeast cell exhibits an abnormal phenotype characterized by elongated buds, nucleated pseudo-hyphae, karyokinetic defects and nuclear fragmentation (Nikitin et al., 2008; Rossi et al., 2010). Moreover, the metabolism of the translocants was severely impaired; they show, in particular, altered fitness on different carbon sources, different sporulation efficiencies and ability to flocculate. The integration of the same cassette at the two target *loci* can be processed in different ways generating strains different in karyotype and consequently in phenotype and physiology. These data suggest that the scrambling of gene regulation throughout the genome triggered by the integration of a linear DNA fragment through recombination is a great force for evolution. Indeed, among the broad panorama of mutants generated by a translocation, few of them, or perhaps only one of them, will be favored in survival and life span, adapting better to new environmental conditions or oxidative stress. That is exactly what happens to the mammalian cells after neoplastic transformation, when genomic defects are translated to phenotypic aberrations and it represents the great plasticity and diversity of cancer cells.

### GENOME-WIDE EFFECTS ON REGULATION OF GENE EXPRESSION

It was demonstrated that the BIT system causes an increased expression of the genes around the breakpoints up to five times (cis effect), coinciding with an increased level of the RNA polymerase II binding to their promoters, and with the pattern of histone acetylation (Nikitin et al., 2008). Furthermore, many other genes not involved in the specific translocation events are deregulated (trans effect). Extensive transcriptome and fluorescence-activated cell sorting (FACS) analysis of the translocant pointed out that the acentric chromosome fragments are duplicated or integrated through micro-homology in the genome and that many cells are blocked in G2/M phase. These results indicate that the translocant cells have adapted to the checkpoint response after the initial DNA damage induced by BIT. More recently, an implementation of the BIT system was created in order to bridge together two homologous chromosomes in a diploid cell (Tosato et al., 2009). In this case, the experiments demonstrated that BIT happens with low frequencies producing LOH and regions of hemizygosity by deletion. The frequency of targeted BIT between homologous chromosomes is lower or the same than between heterologs, supporting the idea that a checkpoint system might actively prevent mitotic LOH in eukaryotic diploid cells. The phenotypic and transcriptional aberrations of the translocant between homologous chromosomes are negligible if compared to those of non-reciprocal translocants between heterologs. Moreover, the quantitative analysis of the expression of several genes around the breakpoints indicated the over-expression of the multi-drug resistance gene VMR1. Remarkably, VMR1 is the budding yeast homolog to the human MRP4, which is highly expressed in LOH-associated types of cancer such as primary neuroblastoma (Norris et al., 2005).

### ANEUPLOIDY

Recently, it was demonstrated that the HRS and the BIR pathway are both responsible for the formation of the initial non-reciprocal translocation and that the proximity of the targeted *loci* with specific genomic elements, such as autonomously replicating sequences (ARS) or repeated DNA regions, may influence not only the efficiency of the event, but also the frequency of secondary rearrangements and aneuploidies (Tosato and Rossi, personal communication).

Aneuploidies are a landmark for cancer, but it is still not completely clear if they are an innocent by-product due to checkpoint gene alterations or a driver of evolutionary processes leading to neoplastic transformation. Further investigations of the molecular players hidden behind the BIT system and responsible for the primary and secondary rearrangements, will shed light to this complex question.

## FUTURE PERSPECTIVES AND CONCLUSIONS

In this short review we analyze some analogies between yeast and cancer cells by the metabolic and genomic point of view. Typical traits of a neoplastic transformation are loss of growth control, the consequential continuous energetic demand and aneuploid conditions due to genome instability. We found that in some translocants, where clear phenotypic defects are visible, there are also important metabolism impairments such as a reduced fitness to grow on glucose-deprived media. Effectively, after a wide proteomic and transcriptomic analysis (Nikitin et al., 2008; Nikitin and Bruschi, personal communication), we found that the trans effect of BIT does not mainly concern, as expected, recombination-related genes, but on the contrary, metabolic genes. From a purely logic point of view, to adapt to a different environmental condition and to evolve (a malignant status is also an evolution) the cells at first must change their own metabolism. In this way, the cellular fitness will be improved and the new mutants will be suddenly ready to overgrow the normal, low life span population. To understand how to stop this amazing ability to adapt and immortalize, we have to use simple single-cell models able to retain an induced aneuploid status and chromosomal alterations like telomere–telomere fusions, typical of many cancers. The ideal organism is *S. cerevisiae* because it has a good amount of chromosomes to play with, the best annotated genome, an ability to survive and grow in haploid and diploid state, a simple switch between fermentation and respiration and a great tolerance to ploidy variations. The phenotypic and metabolic changes observed in *Saccharomyces* after a translocation resemble some of the peculiarities observed in tumorigenesis. Studying metabolism, experiments conducted in yeast are less biased compared to mammalian cell culture, as culture conditions and genetic background have strong influence on the status of the metabolic network. Most routes of central metabolism are strongly conserved between yeast and human, and it appears that the same is true for basic control mechanisms. We have reviewed the regulatory function of yeast and human PK on metabolism, and conclude that this enzyme presents a central and conserved coordinator between energy production, ROS clearance, and amino acid metabolism. Elaborating the principles of the metabolism of rapidly proliferating cells (ROS quantitation, respiratory proficiencies) and extensively studying the altered genetic expression in a collection of different BIT translocants will help finding the effectors to revert, if not the altered karyotype, at least some abnormal phenotype of aneuploid cancerogenic cells. In addition, this straightforward technology could be extrapolated to higher organisms to implement a molecular modeling of spontaneous genome rearrangements leading to speciation in lower eukaryotes or LOH in mammalian cells.

## Conflict of Interest Statement

The authors declare that the research was conducted in the absence of any commercial or financial relationships that could be construed as a potential conflict of interest.
